# Supplementary material for: Establishment of Two Novel Ovarian Tumor Cell Lines with Characteristics of Mucinous Borderline Tumors or Dedifferentiated Carcinoma—Implications for Tumor Heterogeneity and the Complex Carcinogenesis of Mucinous Tumors
Source: Cancers (Basel). 2025 May 20;17(10):1716. doi: 10.3390/cancers17101716 (PMC12110342; doi:10.3390/cancers17101716)
Supplement: Supplementary file 1 [file cancers-17-01716-s001.zip › Supplementary.pdf]

Article

Establishment of two novel ovarian tumor cell lines with characteristics of mucinous borderline tumors or dedifferentiated carcinoma—Implications for tumor heterogeneity and the complex carcinogenesis of mucinous tumors

Hasibul Islam Sohel[1], Umme Farzana Zahan[1], Tohru Kiyono[2], Masako Ishikawa[1], Sultana Razia[3], Kosuke Kanno[1], Hitomi Yamashita[1], Shahataj Begum Sonia[1], Kentaro Nakayama[4,*] and Satoru Kyo[1,*]

Supplementary Table S1. STR profiles of two unique epithelial cell lines originated from the same sample of MBOT.

STR locus	HMucBOT-1	HMucBOT-2	MBOT
AMEL	X	X	X
D3S1358	17	14, 17	14, 17
TH01	6	6	6
D21S11	29, 32.2	29, 32.2	29, 32.2
D18S51	14, 17	14, 17	14, 17
Penta_E	14, 18	14, 18	14, 18
D5S818	11, 12	11, 12	11, 12
D13S317	9	9, 12	9, 12
D7S820	12	12	12
D16S539	8, 12	8, 12	8, 12
CSF1PO	12	12	12
Penta_D	10, 13	10, 13	10, 13
vWA	16	16	16
D8S1179	10, 15	10, 15	10, 15
TPOX	8	8	8
FGA	22, 23	22, 23	22, 23

MBOT, mucinous borderline ovarian tumor.

Supplementary Table S2. Description of primary antibodies.

Antibody Name	Catalog no.	Manufacturer
Pan-Cytokeratin (C11)	sc-8018	Santa Cruz Biotechnology
Cytokeratin 7 (RCK105)	sc-23876	Santa Cruz Biotechnology
PAX8	10336-1-AP	Proteintech
MUC1	MA1-06503	Thermo Fisher Scientific
AE1/AE3	67306	Cell Signaling Technology
CAM5.2	Mob469	Diagnostic BioSystems
Desmin	sc-23879	Santa Cruz Biotechnology
EMA	Z2048MP	Thermo Fisher Scientific
S100	ab34686	Abcam
Twist	ab175430	Abcam
Snail	PA5-23482	Thermo Fisher Scientific
Vimentin	ab92547	Abcam
MLH1	M3639	Dako
MLH6	M3640	Dako
MSH6	M3639	Dako
PMS2	M3647	Dako

Note: The aforementioned antibodies are utilized for immunohistochemistry analyses.

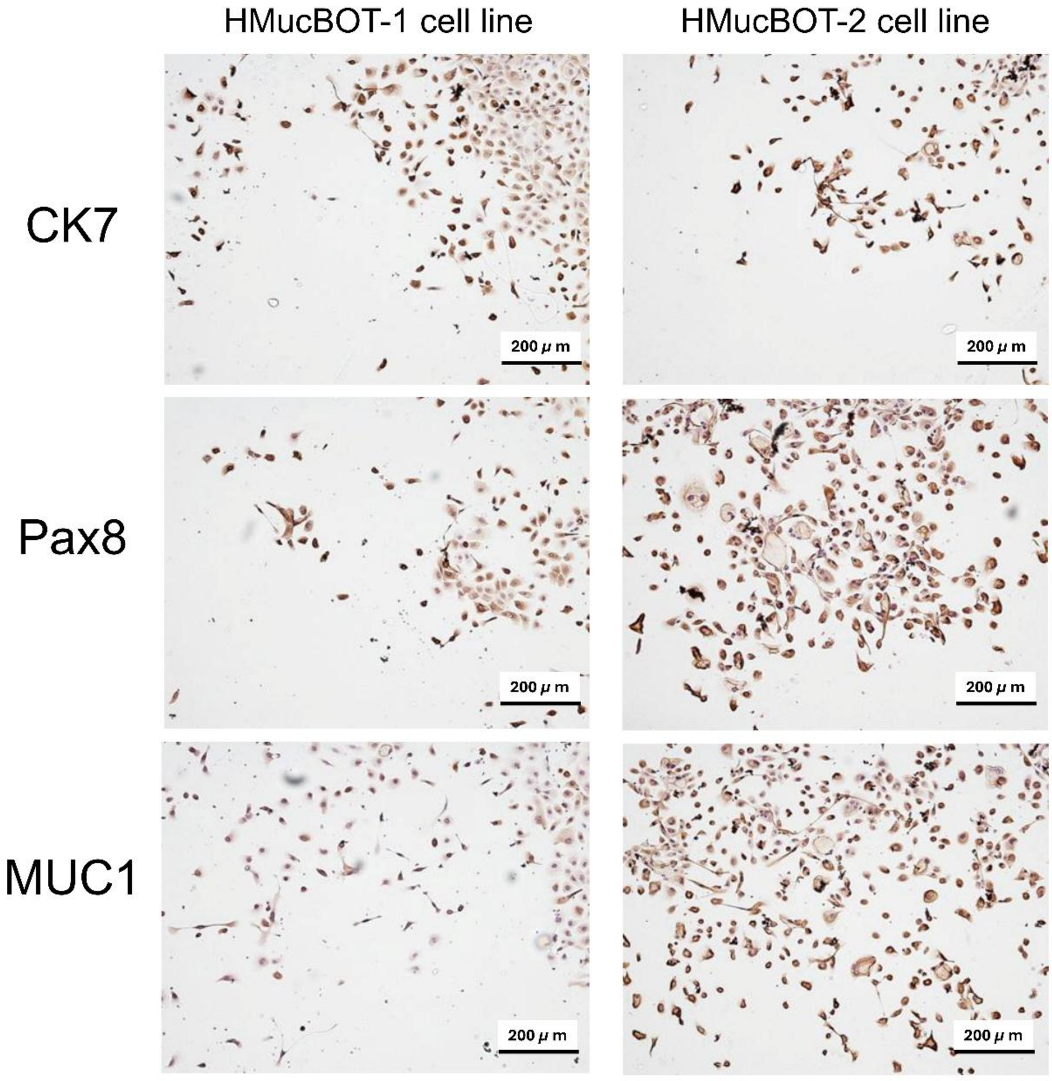

Supplementary Figure S1. HMucBOT-1 and HMucBOT-2 cells showed positive expressions of CK7, PAX8, and MUC1.
